# The Effect of Fluid Shear Stress on the In Vitro Release Kinetics of Sirolimus from PLGA Films

**DOI:** 10.3390/polym9110618

**Published:** 2017-11-15

**Authors:** Quan Zheng, Zhaowei Chu, Xiaoming Li, Hongyan Kang, Xiao Yang, Yubo Fan

**Affiliations:** 1 School of Biological Science and Medical Engineering, Beihang University, Key Laboratory for Biomechanics and Mechanobiology of Ministry of Education, Beijing 100083, China; zhengquan@buaa.edu.cn (Q.Z.); lixm@buaa.edu.cn (X.L.); hongyankang@buaa.edu.cn (H.K.); xiaoyang@buaa.edu.cn (X.Y.); 2 Beijing Advanced Innovation Centre for Biomedical Engineering, Beihang University, Beijing 102402, China; 3 Beijing Key Laboratory of Rehabilitation Technical Aids for Old-Age Disability, National Research Center for Rehabilitation Technical Aids, Beijing 100176, China; czwqz2005@163.com

**Keywords:** sirolimus release, PLGA degradation, uniform shear stress, parallel plate flow chamber, drug-eluting stent, biodegradable coating, hemodynamics

## Abstract

Drug-carrying coatings of stents implanted in blood vessels are exposed to various blood flows. This study investigated the effect of fluid shear stress on the in vitro release kinetics of sirolimus from poly(lactic-*co*-glycolic acid) (PLGA) films. The homemade parallel plate flow chamber was used to exert quantitative shear stress on the sirolimus-carrying film. By adjusting the flow rate of the release media in the chamber, three levels of shear stress (3.6, 12.0, and 36.0 dyn/cm^2^) were respectively applied. For each level of shear stress employed, the release kinetics of sirolimus from the PLGA films exhibited a four-phase profile: an initial burst release phase (Phase I), a lag phase (Phase II), a second burst release phase (Phase III), and a terminal release phase (Phase IV). During Phases I and II, sirolimus was released slowly and in small amounts (<10%); however, during Phases III and IV, the drug release increased considerably. Comparisons of different shear stresses indicated that greater shear stress resulted in earlier and faster sirolimus release, with more cumulative drug release observed. PLGA film degradations (molecular weight reduction, mass loss, and surface topographical variations) were also investigated to better explain the observed drug release behavior. Consequently, fluid shear stress was found to significantly accelerate the release of sirolimus from the PLGA matrices. Therefore, this study could provide a practical method for evaluating the in vitro drug release from polymer matrices under uniform shear stress, and might help improve the design of biodegradable coatings on drug-eluting stents.

## 1. Introduction

Sirolimus, also called rapamycin, is a triene macrolide antibiotic isolated from the culture medium of *Streptomyces hygroscopicus*. Sirolimus has immunosuppressive, antitumor, anti-inflammatory, and anti-restenotic properties; accordingly, it has been widely used in organ transplantation and antitumor treatment [1]. In particular, the implantation of sirolimus-eluting stents has significantly lowered the rate of restenosis after percutaneous coronary intervention, thus achieving an important milestone in heart disease treatment [2,3,4].

A sirolimus-eluting stent generally comprises a bare-metal stent, sirolimus, and a polymer coating. The bare-metal stent expands the vessel to prevent vascular retraction. The polymer coating serves as the sirolimus storage and release control platform, and therefore affects the drug release [5]. Preclinical animal tests and clinical experiments have shown that the treatment effects of drug-eluting stents (DESs) depend directly on their drug release kinetics, which are not only related to the drug properties and the stent geometric configuration, but also related to the coating [6,7]. Early DESs employed non-degradable polymers as coating matrices. However, such materials can induce late thrombosis and hypersensitivity; hence, current DESs mostly use biodegradable materials [8,9,10,11]. Poly(lactic-*co*-glycolic acid) (PLGA) is a Food and Drug Administration (FDA)-approved biodegradable polymer that is widely used in biomedicine. PLGA degrades into non-toxic lactic acid and glycolic acid through hydrolysis in vivo, and these acids then enter the tricarboxylic acid cycle to be further degraded into carbon dioxide and water [12]. Because of its satisfactory biocompatibility, biodegradability, and adjustable erosion time, PLGA has been extensively employed in absorbable sutures [13], tissue engineering scaffolds [14,15], bone surgery [16], and drug delivery systems [17,18,19]. More notably, various kinds of PLGAs have been commonly used as coating materials in commercially available DESs [20,21,22].

When PLGA is employed as a drug carrier, its degradation kinetics directly affect the drug release kinetics. The degradation rate of PLGA not only depends on its own properties, such as the proportion of lactic acid and glycolic acid, molecular weight, crystallinity, and shape, but also depends on other chemical and biological factors, including the degradation media, enzymes, and microorganisms [23,24,25,26]. Moreover, when PLGA is employed in fabricating implants, the local mechanical environments (e.g., cyclic loading) substantially affect the degradation kinetics of PLGA [27,28,29]. Hemodynamic shear stress is the most common type of mechanical load caused by blood flow in the vessels [30]. In addition to affecting the functions of vascular cells, hemodynamic shear stress is closely related to the transportation and concentration distribution of physiologically active substances in vascular beds [31,32,33]. Hemodynamic shear stress has been documented to play a critical role in atherosclerosis and in-stent restenosis [34,35]. Blood flow rates in human arteries vary markedly, and such variations lead to considerably different shear stresses on the drug-carrying coatings of implanted stents [36,37,38]. Our previous studies showed that fluid shear stress significantly affected the degradation rate of PLGA [39,40]. However, to our knowledge, no quantitative studies have confirmed whether fluid shear stress influences the in vitro drug release kinetics from PLGA coatings.

Several approaches have been developed to evaluate the in vitro release kinetics of drugs from stent coatings [41,42,43]. A non-compendial method is to immerse a DES in a small bottle that contains a release medium and to shake it at a specific speed, after which the drug release from the DES is investigated [41]. The shortcoming of this method is that it does not consider the fluid mechanical environment of the DES in the vessel. To compensate for this shortcoming, the United States Pharmacopeia has recommended using a USP Apparatus 4 (Flow-through Cell) or USP Apparatus 7 (Reciprocating Holder) [42,43]. Although both devices can create luminal flow to simulate the intravascular hemodynamic environment, the actual luminal flow field that surrounds a DES is non-uniform. In addition, the complicated geometric configuration of the stent can further lead to a non-uniform distribution of shear stress on the coating, which then causes the drug to be released from various points of the coating at varying rates [44]. Therefore, to quantitatively analyze the effect of shear stress on the drug release kinetics from a stent coating, an experimental method that can apply a uniform fluid shear stress to a drug-carrying coating should be developed.

Two conditions must be simultaneously met to ensure that the drug can be released from the coating under a uniform shear stress: (1) the drug-carrying coating cannot have a complicated geometric configuration, so its surface should be maintained on the same plane; (2) an experimental device that can create a uniform flow field for the drug-carrying coating should be used. To obtain the drug release profile from the coating without accounting for the stent geometric configuration, several researchers have employed drug-carrying films prepared on a plate as a substitute for a DES coating when conducting in vitro release tests [45,46]. Additionally, it has been documented that there is a uniform flow field at the bottom of a parallel plate flow chamber (PPFC) [47,48]. Therefore, we can reasonably assume that replacing a DES with a drug-carrying film and placing the film at the bottom of a PPFC can result in the application of a uniform shear stress to the film. The present study examined the effect of shear stress on the release of sirolimus from PLGA films in uniform flow fields. The home-made PPFCs were used to exert quantitative shear stresses on sirolimus-carrying films. The released amounts of sirolimus over time were measured and used as the basis for comparing the drug release profiles under different shear stresses. The variations in mass, molecular weight, and surface topography of the films were also evaluated to understand the observed drug release behavior. The results of this study might contribute to the design of biodegradable coatings on DESs.

## 2. Materials and Methods

### 2.1. Materials

Sirolimus was supplied by Kerui Pharmaceutical Company (Fuzhou, China). PLGA (LA/GA = 50/50, MW = 126 kDa) was obtained from Jinan Daigang Biomaterial Company (Jinan, China). Ethanol of high-performance liquid chromatography (HPLC) grade was purchased from Sinopharm Chemical Reagent Beijing Company (Beijing, China). Tween-20 was from Amresco Company (Solon, OH, USA). All unspecified chemical reagents were of analytical grade.

### 2.2. Preparation of Sirolimus-Carrying PLGA Films

PLGA films were prepared using a solvent casting method. First, PLGA was completely dissolved in chloroform to reach a concentration of 0.85% (*w*/*v*). Sirolimus (0.15%, *w*/*v*) was added to the PLGA solution and mixed well to form a homogeneous solution of drug and polymer. Then, the mixture (10 mL) was placed on a glass plate (length: 150 mm, width: 50 mm), which was placed on a level-adjusted platform. The sample was kept at room temperature (25 °C) for five days so that the solvent dried naturally. Finally, the sirolimus-carrying PLGA films were placed in a vacuum oven at 37 °C for seven days to evaporate residual chloroform. A micrometer was used to measure the thickness of the dried drug-carrying PLGA films, and the average thickness was found to be 0.11 mm ± 0.01 mm.

### 2.3. Release of Sirolimus

The experimental device that applied a uniform shear stress to the sirolimus-carrying PLGA film was described in our previous paper [39]. Briefly, this apparatus mainly consisted of a self-designed PPFC and a circulating system. The glass plate with the sirolimus-carrying PLGA film was placed at the bottom of the PPFC. A 50 mL aqueous solution of Tween-20 (4 g/L) was used as the release medium, and infused into the circulating system. The sirolimus-carrying PLGA films in this study were divided into three groups by the level of shear stress. In Group L, Group M, and Group H, the flow rates of the release media were set to be 3, 10, and 30 mL/min, respectively, which maintained the levels of fluid shear stress at 3.6 (low), 12.0 (medium), and 36.0 dyn/cm^2^ (high). For each group, four sirolimus-carrying PLGA films were used. The in vitro sirolimus release experiments were performed in a constant temperature oven (37 °C). Every 24 h, the release medium was completely refreshed. The sirolimus content in the release medium was measured using HPLC.

### 2.4. Analysis of Sirolimus

The quantitative analysis of sirolimus was performed using an HPLC system equipped with Waters 600E pumps, and a 2487 dual wavelength absorbance detector (Waters Corp., Milford, MA, USA). The chromatography column employed was a J’sphere ODS-H80 (250 mm × 4.6 mm, YMC Co., Kyoto, Japan), and its temperature was maintained at 60 °C. A mobile phase of water and ethanol (35/65, *v*/*v*) was used at a flow rate of 0.6 mL/min. The ultraviolet detection wavelength was set at 277 nm. Chromatographic peak data collection, analysis, and processing were conducted using Waters Empower software.

### 2.5. Sirolimus-Carrying PLGA Film Degradation

At designated time points, sirolimus-carrying PLGA films were retrieved from the flow chambers, and carefully removed from the glass plates. The samples were then rinsed with distilled water, and dried in a vacuum oven until they had a constant weight. The mass loss of the sirolimus-carrying PLGA film was evaluated using gravimetric analysis:

Weight loss (%) = (*W*_ini_ − *W*_dry_)/*W*_ini_ × 100% (1)

where *W*_ini_ is the initial film mass, and *W*_dry_ is the dried mass of the degraded film.

The PLGA film that had undergone a mass variation analysis was next dissolved in chloroform and filtered. A viscosity test was administered on the filtered solution by using an Ubbelohde viscometer at 25.0 ± 0.1 °C. The viscosity-average molecular weight of PLGA was obtained with the Mark–Houwink equation [49].

The surface morphology of the film was observed by field emission scanning electron microscopy (FE-SEM) (Quanta 250 FEG, FEI Company, Hillsboro, OR, USA). The magnification of the FE-SEM image was 20,000 times. Prior to being observed, each sample was sputtered with gold for better conductivity.

### 2.6. Statistical Analysis

Experimental data are represented by mean and standard deviation (*n* = 4). Statistical comparisons among groups were performed by analysis of variance (ANOVA) and Tukey’s post hoc test using SPSS 17.0 software (SPSS Inc., Chicago, IL, USA, 2008). A *p* < 0.05 was considered statistically significant.

## 3. Results and Discussion

### 3.1. Sirolimus Release Kinetics

**Figure 1:**
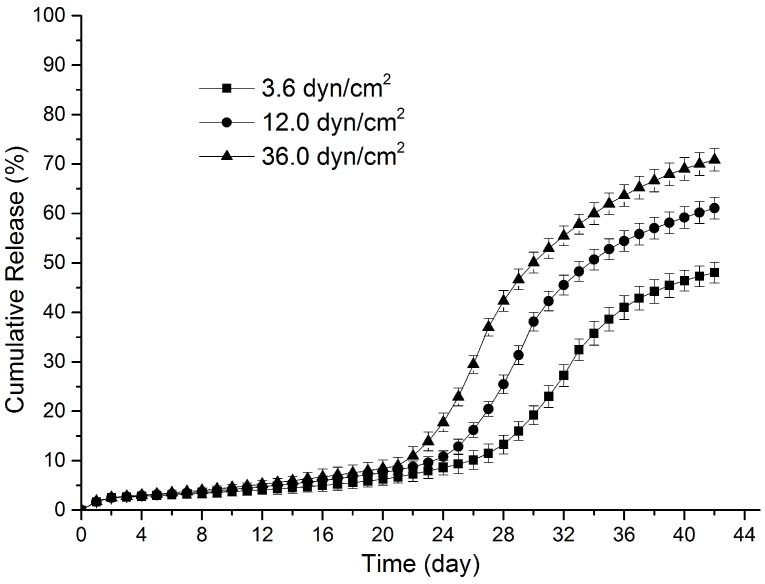
Release curves of sirolimus from poly(lactic-*co*-glycolic acid) (PLGA) films under various shear stresses.

Figure 1 illustrates the release kinetics of sirolimus from PLGA films under various shear stresses. In each group, the sirolimus release kinetics exhibited a four-phase release profile. In Phase I, less than 3% of sirolimus was released in the initial two days. After the small initial burst release, the drug release slowed and continued for an extensive period of time (≥19 days); this duration was also referred to as the lag phase (Phase II). Then, a sudden acceleration of drug release occurred, indicating the completion of the lag phase and the beginning of the second burst release phase (Phase III). During Phase III, the rate and duration of drug release were strongly dependent on the shear stress level. As the remaining drug content in the film decreased, the drug release slowed down, thus signaling the onset of the terminal release phase (Phase IV). To explain the mechanism of the sirolimus release kinetics from the PLGA films, the following zero-order release equation was used to analyze the sirolimus release data during different release phases:

*M*_d_/*M_∞_* = *k*_d_*t* (2)

where *M*_d_/*M_∞_* is the proportion of the drug released at time *t*, and *k*_d_ denotes the constant of the drug release kinetics and can be obtained by linear fitting [50].

According to the fitting results (Table 1), the differences in shear stress resulted in two major changes in the sirolimus release kinetics. (1) Phase II lasted for approximately 21 days in Group L, but for 19 days in Group H, indicating that an increase in shear stress moderately shortened the duration of Phase II, and thus brought forward the relatively earlier onset of Phases III; (2) during Phases III and IV, the drug release increased significantly among the three groups; however, greater shear stress resulted in earlier and faster release of sirolimus from the PLGA films, further causing these two phases to be significantly shorter. For example, the initiation of sirolimus release in Group L during Phase III occurred on Day 24, and proceeded at a rate of 2.97% day^−1^ for 13 days; by contrast, it began in Group H on Day 22 at a higher rate of 5.43% day^−1^, significantly reducing the duration of Phase III to 8 days, and subsequently leading to the earlier beginning of Phase IV. During Phase IV, higher release rates were still observed in Group H than in Group L; thus, it seemed reasonable to infer that the drug release from the films was completed earlier under greater shear stress. On Day 42, the highest (70.9% ± 2.3%), medium (61.1% ± 2.2%), and lowest (48.0% ± 2.1%) cumulative sirolimus release amounts were observed in Groups H, M, and L, respectively (*p* < 0.05, ANOVA). These data further confirmed that sirolimus release from the PLGA matrices was significantly accelerated by shear stress.

### 3.2. Degradation of PLGA Films with Sirolimus

**Figure 2:**
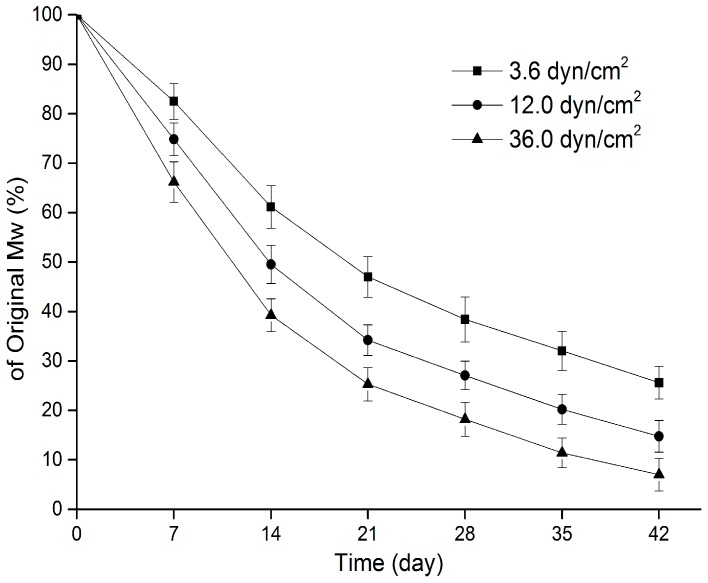
Variations in the molecular weight of sirolimus-carrying PLGA films over time under various shear stresses.

The molecular weight variations of sirolimus-carrying PLGA films are presented in Figure 2. The degradation curves reveal that the molecular weight of all PLGA films decreased as soon as they were immersed in the release medium, and the molecular weight continued to drop during the incubation. Because the reduction in the molecular weight of the PLGA was caused by the random hydrolysis of its ester bond, the PLGA degradation process conformed to the first-order kinetics. Fitting of the PLGA molecular weight and degradation time was then performed using the following equation:

ln(*M*_p_/*M*_0_) = −*k*_p_*t* (3)

where ln is the natural logarithm, *M*_0_ represents the original molecular weight of PLGA, *M*_p_ represents the molecular weight at the degradation time *t*, and *k*_p_ describes the rate constant of the reaction [51]. The fitting results showed that the fastest decrease in the PLGA molecular weight was seen in Group H (0.062 day^−1^), followed by Group M (0.046 day^−1^) and Group L (0.033 day^−1^), suggesting that the degradation of the PLGA matrix was highly sensitive to the changes in the shear stress caused by the flow of the drug release media.

**Figure 3:**
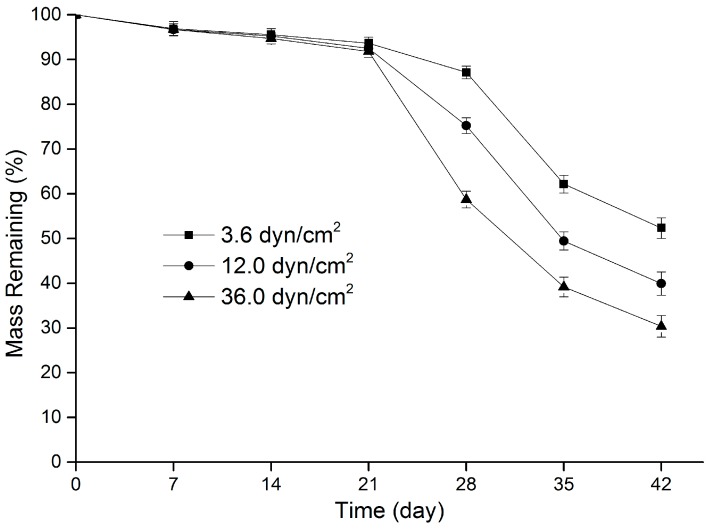
Variations in the mass of sirolimus-carrying PLGA films over time under various shear stresses.

**Figure 4:**
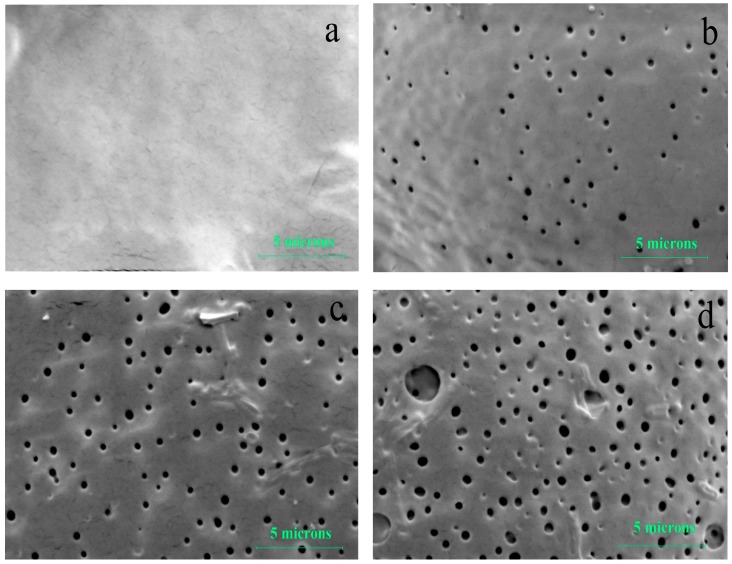
Surface topography of sirolimus-carrying PLGA films under various shear stresses. (**a**) Before degradation. Degradation under shear stress levels of 3.6 dyn/cm^2^ (**b**); 12.0 dyn/cm^2^ (**c**); and 36.0 dyn/cm^2^ (**d**) after 28 days.

As shown in Figure 3, the mass loss of the PLGA films was minimal (<10%) for each group in the first three weeks, and no significant difference in mass loss was identified. In the subsequent three weeks, substantial mass losses were discovered in all films. Moreover, during this period, the rate of mass loss increased significantly with the shear stress magnitude. For example, on Day 28, the average weights in Groups L, M, and H were, respectively, 87.1% ± 1.4%, 75.2% ± 1.8%, and 58.7% ± 1.9% of their original values (*p* < 0.05, ANOVA). The clear connection between shear stress and the degradation of PLGA was further supported by the FE-SEM images of the films on Day 28 (Figure 4). Surface topographical variations indicated that when the shear stress was elevated, more and bigger pores appeared on the films in deeper locations, and some interconnected pores occurred. In all, the above three results (molecular weight reduction, mass loss, and surface change) confirmed that an increase in shear stress resulted in a faster degradation of the PLGA films.

### 3.3. Discussion of Release Profiles

The drug release from PLGA-based delivery systems is controlled by PLGA degradation and numerous other factors. Most drug release curves associated with PLGA matrices belong to one of three types: biphasic [52], triphasic [53], or four-phasic patterns [54]. Previous sections of this paper demonstrate that the mechanical environment in which PLGA is located can significantly affect its degradation. Herein, the degradation process of PLGA films under various shear stresses is discussed to further understand the obtained sirolimus release curves.

A comprehensive analysis of Figure 2 and Figure 3 revealed that the degradation of the PLGA films occurred in three stages, which is consistent with the degradation process of the previous report [55]. In present study, the film variation in each stage was closely related to the characteristics of sirolimus release. In the first stage of polymer degradation, water molecules in the release media directly penetrated the films and caused random breakage of the PLGA ester bonds; hence, a remarkable decrease in the PLGA molecular weight was observed. However, the PLGA chains were too long and the majority of degraded fragments could not escape from the bulk; consequently, a little mass loss was observed in the samples. Accordingly, sirolimus was released slowly and in small quantities (i.e., Phase II of drug release). Notably, during this period, greater shear stress led to a faster reduction in the PLGA molecular weight, which facilitated film mass loss and drug release in the following two stages.

During the second stage, the PLGA molecular weight continued to decrease, causing the formation of some leachable oligomers and their diffusion into the release medium. This led to a significant mass loss, and a substantial amount of sirolimus was also released from the matrix (Phase III of drug release). Moreover, it was particularly noteworthy that greater shear stress could create more leachable oligomers at an earlier time, resulting in earlier and faster film mass loss and drug release. Surface topographical variations identified from FE-SEM images also revealed that greater shear stress led to more and bigger micropores on the film surfaces (Figure 4); the formation of these micropores contributed to drug diffusion and release. In the third stage, oligomer fragments continued to be degraded into completely water-soluble products, during which any residual drug in the PLGA was released (Phase IV of drug release).

In short summary, according to the analysis on the four-phase release profile of sirolimus, except the small initial burst release (generally attributed to surface adhered drugs), the drug release behavior during the next three phases synchronized with the three-stage degradation process of the PLGA films. More importantly, by significantly affecting the degradation rate of PLGA films in each stage, the applied shear stress caused significant variations in the sirolimus release kinetics. Although these findings cannot be used to directly deduce their clinical implications for anti-restenosis treatment, they may provide a basis for the design of DESs. Therefore, research on biodegradable stent coatings may consider the effects of different shear stresses on the degradation of the matrices, as well as the resulting influences on drug release.

### 3.4. Research Limitations

This study has four limitations. First, the degradation of the drug-free PLGA films, which can be helpful for explaining the sirolimus release kinetics, is ignored in this study. Second, the shear stress applied to the sirolimus-carrying film was created by a steady flow. In reality, however, the hemodynamic environment of human arteries in which stents are placed is complicated and diverse; physiological pulsatile flows coexist with steady flow [56,57]. Third, the aqueous solution containing Tween-20 was used as the in vitro release medium. Sirolimus is very unstable in various aqueous solutions and has poor water solubility [58,59,60,61]. According to our previous work, the aqueous solution was supplemented by Tween-20 (4 g/L) to increase the stability and solubility of sirolimus, thereby attaining a favorable sink condition for the sirolimus release experiments [62]. However, the Tween-20 aqueous solution in this research could not really simulate the blood components. Fourth, 50 mL of the release medium was used as the degradation solution of PLGA and refreshed every 24 h. As a result, the significant pH change in the solution was not observed during the period. However, the accumulation of acid degradation products in the living tissues can decrease the local pH, which can further play a substantial impact on polymer degradation and drug release. Therefore, to further understand the release kinetics of sirolimus from PLGA coatings, our future research will conduct related experiments whose conditions may be more similar to the human hemodynamic and blood chemistry environments.

## 4. Conclusions

Overall, this study mainly investigated the in vitro release kinetics of sirolimus from PLGA films in uniform flow fields using PPFCs. Fluid shear stress was found to significantly accelerate sirolimus release. Moreover, the observed drug release profiles were better explained by the film degradation results. Therefore, this study could provide a practical method for evaluating in vitro drug release from polymer matrices under uniform shear stress, and might help improve the design of biodegradable coatings on DESs.

## Figures and Tables

**Table 1:** Release kinetics of sirolimus from poly(lactic-*co*-glycolic acid) (PLGA) films under various shear stresses.

Shear Stress	Phase	Period (Day)	*k*_d_ (% Day^−1^)	*R*^2^
3.6 dyn/cm^2^	Phase I	1–2	ND	-
	Phase II	3–23	0.24	0.953
	Phase III	24–36	2.97	0.960
	Phase IV	37–42	1.03	0.987
12.0 dyn/cm^2^	Phase I	1–2	ND	-
	Phase II	3–22	0.30	0.976
	Phase III	23–32	4.37	0.973
	Phase IV	33–42	1.37	0.972
36.0 dyn/cm^2^	Phase I	1–2	ND	-
	Phase II	3–21	0.34	0.986
	Phase III	22–29	5.43	0.987
	Phase IV	30–42	1.71	0.971

Note: The zero-order release equation was used to fit the various phases in the sirolimus release curves, which provided the release kinetics of sirolimus. *R*^2^ is the goodness-of-fit, and *k*_d_ is the rate constant of the drug release. ND = not done.
